# An Exon-Specific U1snRNA Induces a Robust Factor IX Activity in Mice Expressing Multiple Human FIX Splicing Mutants

**DOI:** 10.1038/mtna.2016.77

**Published:** 2016-10-04

**Authors:** Dario Balestra, Daniela Scalet, Franco Pagani, Malgorzata Ewa Rogalska, Rosella Mari, Francesco Bernardi, Mirko Pinotti

**Affiliations:** 1 Department of Life Sciences and Biotechnology, University of Ferrara, Ferrara, Italy; 2 Internation Centre for Genetic Engineering and Biotechnology, Trieste, Italy; 3 Haemostasis & Thrombosis Center, University of Ferrara, Ferrara, Italy; 4 LTTA Center, University of Ferrara, Ferrara, Italy

**Keywords:** Exon-Specific U1snRNA, hemophilia B, ExSpeU1, mice, splicing

## Abstract

In cellular models we have demonstrated that a unique U1snRNA targeting an intronic region downstream of a defective exon (Exon-specific U1snRNA, ExSpeU1) can rescue multiple exon-skipping mutations, a relevant cause of genetic disease. Here, we explored in mice the ExSpeU1 U1fix9 toward two model Hemophilia B-causing mutations at the 5′ (c.519A > G) or 3′ (c.392-8T > G) splice sites of *F9* exon 5. Hydrodynamic injection of wt-BALB/C mice with plasmids expressing the wt and mutant (hFIX-2G^5′ss^ and hFIX-8G^3′ss^) splicing-competent human factor IX (hFIX) cassettes resulted in the expression of hFIX transcripts lacking exon 5 in liver, and in low plasma levels of inactive hFIX. Coinjection of U1fix9, but not of U1wt, restored exon inclusion of variants and in the intrinsically weak FIXwt context. This resulted in appreciable circulating hFIX levels (mean ± SD; hFIX-2G5′ss, 1.0 ± 0.5 µg/ml; hFIX-8G3′ss, 1.2 ± 0.3 µg/ml; and hFIXwt, 1.9 ± 0.6 µg/ml), leading to a striking shortening (from ~100 seconds of untreated mice to ~80 seconds) of FIX-dependent coagulation times, indicating a hFIX with normal specific activity. This is the first proof-of-concept *in vivo* that a unique ExSpeU1 can efficiently rescue gene expression impaired by distinct exon-skipping variants, which extends the applicability of ExSpeU1s to panels of mutations and thus cohort of patients.

## Introduction

A crucial event in the earliest splicing step involves the recognition of the donor splice site (5′ss) by the U1 small nuclear ribonucleoprotein (U1snRNP), which is driven by base-pair complementarity with the 5′ tail of its RNA component (U1snRNA).^1^ Over years, variants of the U1snRNA have been exploited to manipulate pre-mRNA processing for therapeutic purposes. Modified U1snRNAs have been used to carry Hammerhead ribozymes^2^ or, more frequently, as antisense molecules to induce skipping of pseudo-exons^3^ or, particularly, of defective exons,^4^ the latter hardly applicable to most human disease genes.

Differently, our and other groups extensively exploited the physiological role of the U1snRNA to promote exon inclusion in the presence of exon-skipping mutations,^5,6,7,8,9,10,11,12,13,14,15,16,17^ a relevant cause of severe forms of human genetic disease.^18,19,20^ The first generation of engineered U1snRNA had a modified 5′ tail with increased complementarity to defective 5′ss, and were shown to rescue exon inclusion in several cellular^5,6,7,8,9,10,11,12,13,14,15,16,21^ and also *in vivo*^17^ disease models. However, these U1snRNAs are often tailored on the disease-causing mutation and have the intrinsic risk of off-target effects by recognizing the partially conserved donor splice site^22^ in other splicing units. For this reason, we recently developed a second-generation U1snRNAs, named Exon Specific U1snRNAs (ExSpeU1s), that are designed to recognize intronic, often poorly conserved and thus gene-specific, regions downstream of the affected exon, and shown to efficiently rescue exon-skipping in different disease models,^23,24,25,26,27,28^ Of great importance, in cellular cultures, a unique ExSpeU1 was able to rescue normal splicing from different type of exon-skipping mutations either at the 5′ss or acceptor splice site (3′ss), or within the exon.^23,29^ Although these gene-specificity and activity ExSpeU1 features would significantly extend the applicability of a single therapeutic molecule to panels of mutations and thus cohorts of patients, a key issue when addressing the numerous diseases with heterogeneous mutational patterns (www.hgmd.cf.ac.uk), this effect of ExSpeU1s *in vivo* has not been investigated yet.

In this study, we explored in mice the efficacy of ExSpeU1 toward exon-skipping mutations leading to Hemophilia B (HB) that is a rare X-linked hemorrhagic disorder (1/35,000 males) associated with reduced levels of factor IX (FIX), a key coagulation protein of liver origin.^30^

HB represents a paradigmatic example of human disease with a heterogeneous mutational pattern comprising several splicing mutations, often associated with severe forms,^31^ characterized by strong unmet medical needs. The observation that even small increase of FIX levels (>2%), quantitatively measurable by functional and protein assays in plasma, would significantly ameliorate the clinical phenotype^32^ makes HB an ideal model to investigate innovative therapeutic approaches such as ExSpeU1.

Through the expression in mice of two natural FIX splicing-defective variants at 5′ss or 3′ss associated with severe HB, we show that the selected ExSpeU1 efficiently rescues human FIX (hFIX) splicing in liver and results in the concurrent robust increase of hFIX protein and coagulant activity in plasma.

## Results

**Figure 1:**
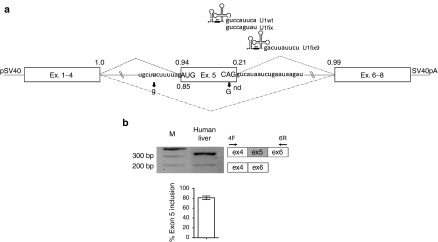
**Features of the human FIX exon 5 contexts and FIX mRNA splicing patterns**. (**a**) Schematic representation of the full-length splicing-competent cassette and of the modified U1snRNA (U1fix and U1fix9) with the 5′ tail sequence placed above the corresponding hFIX target. U1fix9 base pairs with the intronic sequence located 9 nt downstream the exon 5, 5′ss. The U1wt, used as control is also shown for the sake of completeness. Exonic and intronic hFIX sequences are represented by boxes and lines, respectively. Upper and lower dotted lines indicate the normal and aberrant splicing patterns of exon 5. The sequences of the 5′ss and 3′ss splicing junctions, with exonic and intronic regions in upper and lower cases, are also reported together with mutations under investigation (arrows). Scores of the 5′ss and 3′ss, calculated using the Splice Site Prediction by Neural Network tool (http://www.fruitfly.org/seq_tools/splice.html), are reported in normal and mutated conditions. “n.d”, not determined by the computational program as 5′ss. pSV40, SV40 promoter; SV40pA, SV40 poly A signal. (**b**) Evaluation of hFIX alternative splicing patterns in healthy human liver. The schematic representation of the normal and aberrant transcripts, and of primers used for the RT-PCR (arrows) is reported in the right panel. The histogram reports the relative percentage of correct transcripts from three independent liver samples, and results expressed as mean ± SD. Amplified products were separated on 2% agarose gel. M, 100 bp molecular weight marker. RT-PCR, reverse transcription polymerase chain reaction; hFIX, human factor IX; SD, standard deviation.

The *in vivo* ability of ExSpeU1 to restore splicing impaired by exon-skipping mutations was explored in the challenging context of the FIX exon 5 that, as many other splicing units, is characterized by a very weak donor splice site and is intrinsically poorly defined (**Figure 1a**), and indeed prone to aberrant splicing. As a matter of fact, exon 5 is partially skipped even in physiological conditions, as demonstrated by the splicing pattern observed in human liver (**Figure 1b**), and completely excluded from the mature mRNA in the presence of mutations at 5′ss, 3′ss or within the exon, as indicated by cellular models.^23,29^ Among these severe HB causing mutations, the c.519A > G and c.392-8T > G changes at the 5′ss or 3′ss, respectively, were chosen as models to assess in mice the active ExSpeU1 (U1fix9) targeting the intronic sequence starting at position +9 (**Figure 1a**), which has been selected through scanning for efficacy in minigene assays within a panel of ExSpeU1 designed toward the region downstream of the 5′ss of exon 9.^23^

### The U1fix9 significantly rescues exon 5 inclusion of splicing-defective hFIX variants

**Figure 2:**
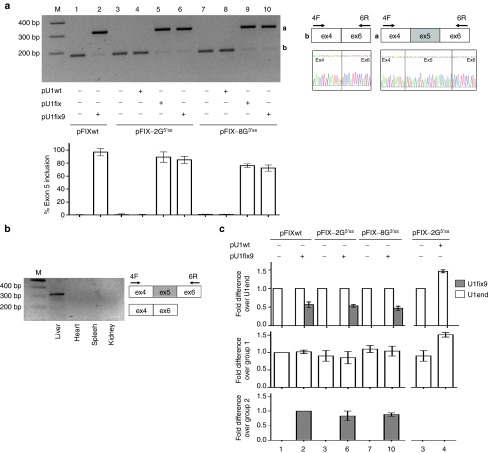
**Evaluation of the U1fix9-mediated rescue of hFIX mRNA splicing in mice**. (**a**) Representative example of hFIX alternative splicing patterns in liver from BALB/c mice injected with the pFIX variants alone (−) or in combination (+) with a molar excess (1.5X) of the pU1wt, pU1fix or pU1fix9. The schematic representation of the normal and aberrant transcripts, and of primers used for the RT-PCR (arrows), is reported in the right panel. Amplified products were separated on 2% agarose gel. M, 100 bp molecular weight marker. The histograms report the relative percentage of correct transcripts in liver from mice injected at the different conditions (*n* = 4 for group 1–2, 4–5, 7–10; *n* = 5 and *n* = 7 for groups 3 and 6, respectively), and results are expressed as mean ± SD. (**b**) Representative example of hFIX splicing patterns in various organs of mice coinjected with pFIX-2G^5′ss^ and pU1fix9. (**c**) Evaluation (middle and lower panels) and comparison (upper panel) of the expression of the endogenous (U1end, white histograms) and ExSpeU1 (U1fix9, gray histograms) by qPCR in liver from mice treated as in panel A. For qPCR, the results are expressed as mean ± SD from three mice for each condition. Values are normalized for the mouse GAPDH and are indicated as fold increase compared to the reference indicated in the Y-axis, set to 1. No transcripts were detected in samples without reverse transcriptase. ExSpeU1, Exon-specific U1snRNA; hFIX, human factor IX; qPCR, quantitative PCR; RT-PCR, reverse transcription polymerase chain reaction; SD, standard deviation.

To deliver in mouse liver the plasmids harboring the hFIX splicing-competent (pFIXwt, pFIX-2G^5′ss^, and pFIX-8G^3′ss^) and the U1snRNA (pU1fix9, pU1fix, and pU1wt) cassettes (**Figure 1a**) we exploited the hydrodynamic tail-vein injection of a high volume of concentrated DNA. This physical method takes advantage of the high-pressure gradient created in liver capillaries, which enhances endothelial and parenchymal cell permeability to DNA. The injection (1.5 µg/g of mouse body weight) of the pFIXwt, pFIX-2G^5′ss^ and pFIX-8G^3′ss^ variants resulted in mouse liver in appreciable hFIX mRNA expression characterized, for the three expression cassettes, by hFIX transcripts lacking exon 5 (**Figure 2a**, lanes 1, 3, and 7). The hFIX mRNA was not detected in other tissues such as heart, kidney, and spleen (**Figure 2b**).

Codelivery of the pU1fix9 with pFIX variants restored correct splicing of FIXwt (from negligible to 97 ± 6%, mean ± SD) and substantially rescued HB-causing variants with impaired 5′ss or 3′ss (from negligible to 85 ± 5% and 72 ± 4% for pFIX-2G^5′ss^ and pFIX-8G^3′ss^, respectively). Comparable results were obtained with the coinjection of the first generation pU1fix, used as control and having perfect complementarity to the wild type FIX exon 5, 5′ss (**Figure 2a**). Conversely, coinjection of the pU1wt, mimicking the endogenous human or mouse U1snRNA, was ineffective.

We performed quantitative polymerase chain reaction (qPCR) with U1fix9-specific primers to assess the expression in liver of the U1fix9, which was clearly detectable only in mice injected with the pU1fix9 (**Figure 2c**; groups 2, 6, and 10), and accounted for approximately half of the endogenous U1snRNA (U1end). Consistently, in mice injected with the pU1wt, the U1snRNA expression showed an apparent increase (1.40 over untreated mice; group 4).

### The U1fix9 significantly increases circulating hFIX levels that results in remarkable improvement of FIX coagulant activity

**Figure 3:**
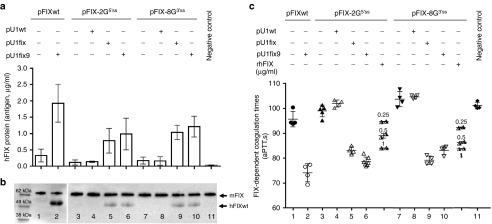
**Evaluation of U1fix9-mediated rescue of hFIX protein and coagulant properties in mice.** (**a**) Plasma hFIX antigen levels (µg/ml) in mice injected with the pFIX variants alone (−) or in combination (+) with a molar excess (1.5X) of pU1wt, pU1fix or pU1fix9. Negative controls are represented by mice injected with saline solution alone. To evaluate hFIX antigen in mouse plasma, samples were diluted 1:10 or 1:20 and evaluated in duplicate. A standard curve was created by adding known amounts of hFIX to mouse plasma, and the sensitivity threshold was 0.40 ng/ml of hFIX. Histograms reports the results obtained in the different mouse groups (*n* = 4, for group 1–2, 4–5, and 7–10; *n* = 5 and *n* = 7, for groups 3 and 6, respectively) and are expressed as mean ± SD. (**b**) Western Blotting analysis performed with the anti-hFIX antibody of the hFIX isoforms in plasma from mice treated as in panel **a**. mFIX, mouse FIX; hFIXwt, human hFIX. (**c**) FIX-dependent activated partial thromboplastin time (aPTT) values (s) in plasma from mice treated as in panel A or in plasma from mice (two independent) expressing the mutated hFIX variants supplemented with known amounts of purified rhFIX (0.25, 0.5 or 1 µg/ml). All samples have been tested in duplicate and the mean reported as single symbols. hFIX, human factor IX; rhFIX, recombinant human FIX.

The hFIX splicing-competent cassettes were designed to incorporate the complete hFIX coding sequence (**Figure 1a**), which enabled us to evaluate the hFIX protein secreted in mouse plasma. Expression of the pFIXwt, pFIX-2G^5′ss^ , and pFIX-8G^3′ss^ produced low hFIX antigen levels (0.33 ± 0.2, 0.12 ± 0.08, and 0.18 ± 0.11 µg/ml, respectively)(**Figure 3a**).

Western Blotting was then exploited to investigate the circulating hFIX. In our experimental set-up, the anti-hFIX antibody recognized both human and mouse FIX, which however are distinguishable by size (**Figure 3b**). Investigations in plasma from the pFIXwt, pFIX-2G^5′ss^ and pFIX-8G^3′ss^ injected mice (lanes 1, 3, and 7) did not reveal appreciable amounts of the normal hFIX.

When the FIX coagulant activity was assessed (**Figure 3c**) we did not appreciate a shortening of the FIX-dependent coagulation times (pFIXwt 96 ± 3 seconds; pFIX-2G^5′ss^, 99 ± 2 seconds; pFIX-8G^3′ss^, 104 ± 3 seconds), as compared with control mice (101 ± 2 seconds). Coinjection of the pU1fix9 with pFIXwt or variants resulted in a significant increase of hFIX antigen levels (pFIXwt 1.9 ± 0.58 µg/ml, *P* < 0.0006; pFIX-2G^5′ss^ 0.99 ± 0.48 µg/ml, *P* < 0.001; and pFIX-8G^3′ss^ 1.2 ± 0.32 µg/ml, *P* < 0.02)(**Figure 3a**) with the concurrent appearance of the full-length hFIX (**Figure 3b**). This led to a remarkable and statistically significant shortening of FIX-dependent coagulation times (*P* < 0.0001), with values in mice expressing the hFIX variants (pFIX-2G^5′ss^, 79 ± 2 seconds; pFIX-8G^3′ss^, 83 ± 2 seconds) resembling those for FIXwt (74 ± 4 seconds) (**Figure 3c**). Similar results were obtained by coinjection of the pU1fix, with a strong increase of hFIX antigen levels (pFIX-2G5′ss, 0.78 ± 0.38 µg/ml; pFIX-8G3′ss 1.0 ± 0.22 µg/ml), the appearance of the full-length isoform and a significant (*P* < 0.0001) shortening in the coagulation time (pFIX-2G^5′ss^, 83 ± 1 seconds; pFIX-8G^3′ss^, 79 ± 2 seconds). Conversely, coexpression of hFIX variants with the U1wt was ineffective.

To provide a quantitative evaluation of the contribution of the rescued hFIX to coagulation times, we spiked plasma from mice expressing the FIX-2G^5′ss^ or FIX-8G^3′ss^ variants only with 1, 0.5 or 0.25 µg/ml of recombinant hFIX (**Figure 3c**), with the lowest concentration shortening the FIX-dependent coagulation times of 5 seconds in set up experiments. Noticeably, in plasma from mice coexpressing the hFIX variants and the U1fix9 the coagulation times was similar or shorter to those obtained by supplementation with 1 µg/ml of recombinant human FIX.

### The active U1fix9 does not act through an antisense mechanism

**Figure 4:**
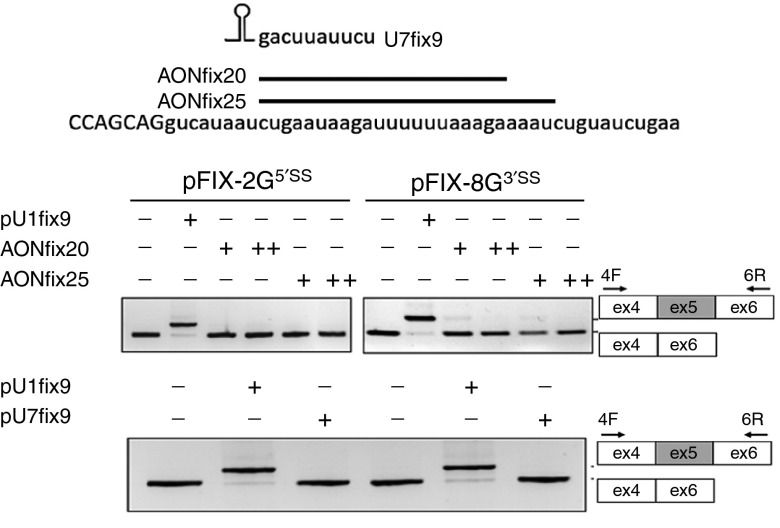
**Evaluation of the U1fix9-mediated mechanism.** Schematic representation of the human F9 exon/intron 5 sequence (in bold and lowercase, respectively) and of antisense oligoribonucleotides (AONfix20 and AONfix25) and of the U7fix9 masking the intronic region recognized by the U1fix9. The gels report the evaluation on alternative splicing patterns in cells transfected with pFIX variants alone (−) or in combination with 1 mmol/l (+) and 100 mmol/l (++) of the AONfix20 and AONfix25, or with a molar excess (1.5X) of the pU1fix9 or pU7fix9. The schematic representation of the normal and aberrant transcripts, and of primers used for the RT-PCR (arrows), is reported in the right panel. Amplified products were separated on 2% agarose gel. RT-PCR, reverse transcription polymerase chain reaction.

To investigate whether the U1fix9 acts by masking an intronic splicing silencer we used two well-established antisense molecules, namely antisense oligoribonucleotides and the U7 snRNA particle,^33^ which were designed to bind to the U1fix9 target sequence (AONfix20, AONfix25, and U7fix9). As shown in **Figure 4**, in cells cotransfected with FIX minigenes harboring the model exon-skipping mutations and the antisense molecules did not result, at variance from the U1fix9, in appreciable exon inclusion. The cellular models also prompted us to evaluate the splicing patterns of five putative U1fix9 off-target genes (*ADRGE 2*; *TANC2*; *PLEKHB1*; *COL21A1*, and *TNIP3* genes) that were predicted by a megaBLAST analysis on the human genome and transcripts database (Human genomic plus transcript; Human G+T) using the 5′tctgaataagat3 ′ sequence harboring the U1fix9 target. The reverse transcription PCR (RT-PCR) analysis in human embryonic kidney (HEK293) transfected with the pU1fix9 did not reveal significant splicing changes for all genes displaying an appreciable expression (see **Supplementary Figure S1**).

## Discussion

In this study, by using splicing-defective hFIX expression as model, we evaluated the *in vivo* efficacy of ExSpeU1s toward multiple mutations, a RNA-based approach that could represent a powerful alternative to replacement gene therapy, especially when the regulation and/or the size of the affected gene make it hardly feasible. The U1snRNA-based technology acts at post-transcriptional levels thus maintaining the transcriptional gene regulation in physiologic tissues, and takes advantage of a very small therapeutic transgene (~600 bp) that can be packaged in virtually any viral vector successfully exploited for gene therapy purposes.^34^ Furthermore, the ExSpeU1 expression is driven by its strong ubiquitous promoter, and in the model of Spinal Muscular Atrophy a single ExSpeU1 copy was shown to trigger an appreciable rescue,^25,28^ which could lead to the use of low doses of viral vector to achieve a therapeutic impact.

On the other hand, we are aware that ExSpeU1, like other personalized approaches based on the mutation type, must cope with the fact that many diseases, such as HB, have highly heterogeneous mutational patterns. In fact, skipping of a given exon is a relatively frequent pathogenic mechanism that can be caused by several different mutations either at the 5′ss or 3′ss, or within the exon, which could weaken the applicability of the modified U1snRNA.

Here, we explored a single ExSpeU1 (U1fix9) toward two model exon-skipping mutations at the opposite 5′ss (c.519A>G) or 3′ss (c.392-8T>G) positions in the FIX exon 5 context. The inability of antisense oligoribonucleotides and modified U7snRNA masking the U1fix9 target sequence to rescue exon 5 inclusions indicated that the U1fix9 is not acting through an antisense mechanism on a functional intronic negative regulatory element. Moreover, we have very recently demonstrated that the U1fix9, as well as other active ExSpeU1s in different human disease models, binds to the intronic sequence downstream of the 5′ss and results in the assembly of a target-specific therapeutic U1-like particle.^28^ This modified U1snRNP restores the spliceosomal activity on the skipped exons, mainly through its associated 70K protein and stem loop IV, an effect that does not require the endogenous U1 snRNP.

To assess the ability of ExSpeU1 in restoring splicing in mice, we exploited the hydrodynamic injection to drive the transient expression of the splicing-defective hFIX cassettes harboring the model mutations and of U1fix9. Interestingly, even in the presence of the nonliver specific SV40 promoter, the hydrodynamic tail-vein injection approach guaranteed an appreciable delivery of plasmid DNA with an appreciable expression of the hFIX transgene mainly in liver. It is worth nothing that species-specific nucleic acid amplification and immunologic assays, distinguishing hFIX from murine FIX, enabled us to evaluate the hFIX in wild-type mice without the confounding effects of the endogenous murine FIX, as indicated by the undetectable hFIX transcripts and protein in untreated mice. Moreover, activated partial thromboplastin time-based coagulation assays were optimized to assess the additive effect of hFIX to the endogenous murine one. Overall, these features provided us with an informative phenotype after treatment and thus a straightforward strategy to overcome the lack of mouse models for the HB mutations under investigation.^35^

The use in mice of the strong SV40 promoter instead of the physiological one might have further promoted exon-skipping of the minigene, which contains a poorly defined exon. As a matter of fact, mRNA splicing and RNA polymerase II elongation rate/processivity are intricately intertwined, with a highly elongating pol II (SV40 promoter) favoring exon exclusion, and *vice versa*.^36^ Notwithstanding the unfavorable exon 5 context, the coexpression of the ExSpeU1 U1fix9, as clearly demonstrated by U1fix9-specific PCR, was able to remarkably recover exon inclusion in the FIXwt and, most importantly, in the presence of the defective 5′ss or 3′ss. Moreover, the strong rescue effect was comparable to that of the first generation U1fix directly targeting the 5′ss. Conversely, the U1wt was ineffective, thus highlighting the crucial role of the engineered U1snRNA 5′s tail in defining both target recognition and splicing correction.

A mandatory issue to evaluate the therapeutic potential of an approach is to assess the impact on protein levels and particularly on the activity in the affected pathway. Consistently, in mice treated with the pU1fix9, we measured a remarkable increase of circulating hFIX protein, and the appearance of the full-length protein isoform. Most importantly, this resulted in a robust shortening (15–20 seconds) in the coagulation times produced by the increase of hFIX protein endorsed of a normal specific activity. From this observation, it is tempting to speculate that the rescue extent would be beneficial if translated in the severe HB patients in whom the therapeutic threshold is rather low.

A second mandatory issue is represented by the specificity of the therapeutic molecules toward the pre-mRNA target, which is hardly predictable by computational tools. With the limitations of not taking into account the functional relevance of incomplete base pairing of the U1snRNA with pre-mRNA, and particularly the crucial interplay with the several splicing factors needed for the assembly of a functional spliceosome, our bioinformatics analysis returned five candidate off-target mRNAs. Interestingly, the RT-PCR investigation in a human cell line transfected with the U1fix9 did not reveal a major impact on their splicing pattern. Although these data need to be extended by high-throughput RNA-seq, they appear to be consistent with our very recent data obtained with ExSpeU1 in the mouse model of Spinal Muscular Atrophy, which revealed gene expression changes in only 12 off-targets genes.^28^ Overall these observations support gene-specific features of ExSpeU1s, which would favor the translation into clinic.

Taken together these findings indicated that multiple HB splicing-defective variants causing exon-skipping could be efficiently rescued *in vivo* by a single ExSpeU1, able to promote appreciable synthesis of hFIX endorsed of a normal coagulant activity.

In conclusion, albeit our mouse models permit the assessment of the impact on coagulation phenotype and not on provoked bleeding, which will require the creation of splicing-specific HB mouse models and ExSpeU1 delivery through adeno-associated viruses,^17^ and despite the unfavorable FIX exon 5 definition, these data provide the first proof-of-concept about the *in vivo* properties of ExSpeU1s, which substantially extends their applicability.

## Materials and methods

*Minigene assays.* Expression vectors, transfections and RT-PCR were conducted as previously described.^23^

Modified U7 snRNA was created by PCR amplification of U7SmOPT vector using FIXsh9U7 (5′ACAGAGGCCTTTCCGCAAATCTTATTCAATTTTTGG3 ′) and Sp6 (5′ATTTAGGTGACACTATAG3 ′) primers. PCR products were digested with *Hin*dIII and *Stu*I and ligated into *Hin*dIII/*Stu*I sites of the U7SmOPT vector. Clones were verified by the sequence analysis. ASOfix20 (5′ucuuuaaaaaaucuuauuca3′) and ASOfix25 (5′gauuuucuuuaaaaaaucuuauuca3′) contain 2′-O-methyl modified ribonucleotides and full-length phosphorothioate backbone.^37^

*Expression vectors, delivery in mice and sampling.* Expression vectors for the (i) splicing-competent hFIX cDNA cassette, either wild-type (pFIXwt) or mutated (pFIX-2G^5′ss^; pFIX-8G^3′ss^), or ii) the U1snRNA, either wild-type (pU1wt) or engineered (pU1fix9, pU1fix) were previously reported.^23^

Eight-week-old BALB/C mice were injected hydrodynamically injected with plasmid DNA in 2.5 ml phosphate buffer saline (PBS) through the tail vein. Blood samples were collected from the retro-orbital plexus into 3.8% sodium citrate 48 hours postinjection. Upon sacrifice, total RNA was isolated from random sections of mouse liver using TRIZOL reagent (Life Technologies, Carlsbad, CA). All procedures were approved and conducted under the guidelines established by the Italian Ministry of Health.

*Evaluation of hFIX mRNA and U1 expression.* Two micrograms of RNA from mouse tissues were retro-transcribed using SuperScript III Reverse Transcriptase (Life Technologies, Carlsbad, CA) with random primers and subsequently PCR amplified with hFIX-specific primers 5′ATTCCTATGAATGTTGGTGTCCCT3′ (4F) and 5′GGGTGCTTTGAGTGATG TTATCCAA3′ (6R). The PCR was run for 40 cycles of 95°C for 30 seconds, 60°C for 20 seconds, and 72°C for 30 seconds. Evaluation of U1snRNA expression was carried out using U1 specific primers 5′AGATCTGATACTTACCTG3′ (U1wtF), 5′AGATCTCATTCTTATTCAG3′ (U1fix9F), and 5′GAACGCAGTCCCCCACTACCAC3′ (U1R). The efficiency of the Syber-Green (Bio-Rad Laboratories, Redmond, WA) based qPCR reactions for U1wt and U1fix9 has been calculated by using U1snRNA-encoding plasmid DNA and the following equation: Efficiency (*E*) = 10^(−1/slope)^. A melting curve analysis showing a single, product specific melting temperature was performed to document the specificity of qPCR reactions. The quantification of U1snRNA was normalized for the mouse GAPDH. qPCR reactions for U1 and mouse GAPDH were carried out to 56°C and 60°C, respectively. Relative expression ratio of U1snRNAs was calculated based on *E* and *Cp* variation following the equation: *R* = (*E*_target_)^Δ*C*p target^/(*E*_ref_)^Δ*C*p^ (ref. 38).

*Assessment of hFIX antigen, protein isoforms, and coagulant activity.* Human FIX antigen levels and isoforms in plasma were evaluated by enzyme-linked immunosorbent assay (ELISA) (Affinity Biologicals, Ancaster, Canada) and Western blotting exploiting a polyclonal anti-hFIX antibody (GAFIX-AP; Affinity Biologicals, Ancaster, Canada).

FIX coagulant activity was assessed by coagulation assays based on the activated partial thromboplastin time. Assays with serial dilution of mouse plasma spiked with recombinant hFIX were conducted to optimize the protocol and magnify the additive effect of hFIX to the endogenous murine one. In the final setting, mouse plasma was diluted 1:200 in Imidazole buffer and mixed 1:1 with hFIX-deficient plasma (George King, Bio-Medical, Overland Park, KS) before adding the activator SynthASil reagent (HemosIL, Instrumentation Laboratory, Lexington, MA) and calcium. Coagulation times were recorded by the ACLTOP 700 instrument (HemosIL, Instrumentation Laboratory).

*Statistical analysis.* Statistical differences among levels were evaluated by a Student′s *t*-test with a *P* < 0.05 considered significant.

**SUPPLEMENTARY MATERIAL** **Figure S1.** Evaluation of alternative splicing patterns of putative off-target mRNAs of U1fix9 in HEK293 cells.

## References

[bib1] Roca, X, Krainer, AR and Eperon, IC (2013). Pick one, but be quick: 5′ splice sites and the problems of too many choices. Genes Dev 27: 129–144. 23348838 10.1101/gad.209759.112 PMC3566305

[bib2] Michienzi, A, Prislei, S and Bozzoni, I (1996). U1 small nuclear RNA chimeric ribozymes with substrate specificity for the Rev pre-mRNA of human immunodeficiency virus. Proc Natl Acad Sci USA 93: 7219–7224. 8692972 10.1073/pnas.93.14.7219 PMC38963

[bib3] Gorman, L, Mercatante, DR and Kole, R (2000). Restoration of correct splicing of thalassemic beta-globin pre-mRNA by modified U1 snRNAs. J Biol Chem 275: 35914–35919. 10969081 10.1074/jbc.M006259200

[bib4] Cazzella, V, Martone, J, Pinnarò, C, Santini, T, Twayana, SS, Sthandier, O et al. (2012). Exon 45 skipping through U1-snRNA antisense molecules recovers the Dys-nNOS pathway and muscle differentiation in human DMD myoblasts. Mol Ther 20: 2134–2142. 22968481 10.1038/mt.2012.178 PMC3498801

[bib5] Baralle, M, Baralle, D, De Conti, L, Mattocks, C, Whittaker, J, Knezevich, A et al. (2003). Identification of a mutation that perturbs NF1 agene splicing using genomic DNA samples and a minigene assay. J Med Genet 40: 220–222. 12624144 10.1136/jmg.40.3.220 PMC1735390

[bib6] Susani, L, Pangrazio, A, Sobacchi, C, Taranta, A, Mortier, G, Savarirayan, R et al. (2004). TCIRG1-dependent recessive osteoporosis: mutation analysis, functional identification of the splicing defects, and *in vitro* rescue by U1 snRNA. Hum Mutat 24: 225–235. 15300850 10.1002/humu.20076

[bib7] Pinotti, M, Rizzotto, L, Balestra, D, Lewandowska, MA, Cavallari, N, Marchetti, G et al. (2008). U1-snRNA-mediated rescue of mRNA processing in severe factor VII deficiency. Blood 111: 2681–2684. 18156490 10.1182/blood-2007-10-117440

[bib8] Pinotti, M, Balestra, D, Rizzotto, L, Maestri, I, Pagani, F and Bernardi, F (2009). Rescue of coagulation factor VII function by the U1+5A snRNA. Blood 113: 6461–6464. 19387004 10.1182/blood-2009-03-207613

[bib9] Tanner, G, Glaus, E, Barthelmes, D, Ader, M, Fleischhauer, J, Pagani, F et al. (2009). Therapeutic strategy to rescue mutation-induced exon skipping in rhodopsin by adaptation of U1 snRNA. Hum Mutat 30: 255–263. 18837008 10.1002/humu.20861

[bib10] Hartmann, L, Neveling, K, Borkens, S, Schneider, H, Freund, M, Grassman, E et al. (2010). Correct mRNA processing at a mutant TT splice donor in FANCC ameliorates the clinical phenotype in patients and is enhanced by delivery of suppressor U1 snRNAs. Am J Hum Genet 87: 480–493. 20869034 10.1016/j.ajhg.2010.08.016 PMC2948791

[bib11] Glaus, E, Schmid, F, Da Costa, R, Berger, W and Neidhardt, J (2011). Gene therapeutic approach using mutation-adapted U1 snRNA to correct a RPGR splice defect in patient-derived cells. Mol Ther 19: 936–941. 21326217 10.1038/mt.2011.7 PMC3098652

[bib12] Sánchez-Alcudia, R, Pérez, B, Pérez-Cerdá, C, Ugarte, M and Desviat, LR (2011). Overexpression of adapted U1snRNA in patients' cells to correct a 5′ splice site mutation in propionic acidemia. Mol Genet Metab 102: 134–138. 21094621 10.1016/j.ymgme.2010.10.013

[bib13] Schmid, F, Glaus, E, Barthelmes, D, Fliegauf, M, Gaspar, H, Nürnberg, G et al. (2011). U1 snRNA-mediated gene therapeutic correction of splice defects caused by an exceptionally mild BBS mutation. Hum Mutat 32: 815–824. 21520335 10.1002/humu.21509

[bib14] Schmid, F, Hiller, T, Korner, G, Glaus, E, Berger, W and Neidhardt, J (2013). A gene therapeutic approach to correct splice defects with modified U1 and U6 snRNPs. Hum Gene Ther 24: 97–104. 23075156 10.1089/hum.2012.110

[bib15] Matos, L, Canals, I, Dridi, L, Choi, Y, Prata, MJ, Jordan, P et al. (2014). Therapeutic strategies based on modified U1 snRNAs and chaperones for Sanfilippo C splicing mutations. Orphanet J Rare Dis 9: 180. 25491247 10.1186/s13023-014-0180-y PMC4279800

[bib16] Balestra, D, Barbon, E, Scalet, D, Cavallari, N, Perrone, D, Zanibellato, S et al. (2015). Regulation of a strong F9 cryptic 5'ss by intrinsic elements and by combination of tailored U1snRNAs with antisense oligonucleotides. Hum Mol Genet 24: 4809–4816. 26063760 10.1093/hmg/ddv205 PMC4527485

[bib17] Balestra, D, Faella, A, Margaritis, P, Cavallari, N, Pagani, F, Bernardi, F et al. (2014). An engineered U1 small nuclear RNA rescues splicing defective coagulation F7 gene expression in mice. J Thromb Haemost 12: 177–185. 10.1111/jth.12471 PMC4238797 24738135

[bib18] Faustino, NA and Cooper, TA (2003). Pre-mRNA splicing and human disease. Genes Dev 17: 419–437. 12600935 10.1101/gad.1048803

[bib19] Buratti, E, Chivers, M, Královicová, J, Romano, M, Baralle, M, Krainer, AR et al. (2007). Aberrant 5′ splice sites in human disease genes: mutation pattern, nucleotide structure and comparison of computational tools that predict their utilization. Nucleic Acids Res 35: 4250–4263. 17576681 10.1093/nar/gkm402 PMC1934990

[bib20] Baralle, D, Lucassen, A and Buratti, E (2009). Missed threads. The impact of pre-mRNA splicing defects on clinical practice. EMBO Rep 10: 810–816. 19648957 10.1038/embor.2009.170 PMC2726684

[bib21] Pinotti, M, Bernardi, F, Dal Mas, A and Pagani, F (2011). RNA-based therapeutic approaches for coagulation factor deficiencies. J Thromb Haemost 9: 2143–2152. 21854538 10.1111/j.1538-7836.2011.04481.x

[bib22] McManus, CJ and Graveley, BR (2011). RNA structure and the mechanisms of alternative splicing. Curr Opin Genet Dev 21: 373–379. 21530232 10.1016/j.gde.2011.04.001 PMC3149766

[bib23] Fernandez Alanis, E, Pinotti, M, Dal Mas, A, Balestra, D, Cavallari, N, Rogalska, ME et al. (2012). An exon-specific U1 small nuclear RNA (snRNA) strategy to correct splicing defects. Hum Mol Genet 21: 2389–2398. 22362925 10.1093/hmg/dds045 PMC3349419

[bib24] Dal Mas, A, Fortugno, P, Donadon, I, Levati, L, Castiglia, D and Pagani, F (2015). Exon-specific U1s correct SPINK5 exon 11 skipping caused by a synonymous substitution that affects a bifunctional splicing regulatory element. Hum Mutat 36: 504–512. 25665175 10.1002/humu.22762

[bib25] Dal Mas, A, Rogalska, ME, Bussani, E and Pagani, F (2015). Improvement of SMN2 pre-mRNA processing mediated by exon-specific U1 small nuclear RNA. Am J Hum Genet 96: 93–103. 25557785 10.1016/j.ajhg.2014.12.009 PMC4289686

[bib26] Nizzardo, M, Simone, C, Dametti, S, Salani, S, Ulzi, G, Pagliarani, S et al. (2015). Spinal muscular atrophy phenotype is ameliorated in human motor neurons by SMN increase via different novel RNA therapeutic approaches. Sci Rep 5: 11746. 26123042 10.1038/srep11746 PMC4485234

[bib27] van der Woerd, WL, Mulder, J, Pagani, F, Beuers, U, Houwen, RH and van de Graaf, SF (2015). Analysis of aberrant pre-messenger RNA splicing resulting from mutations in ATP8B1 and efficient *in vitro* rescue by adapted U1 small nuclear RNA. Hepatology 61: 1382–1391. 25421123 10.1002/hep.27620

[bib28] Rogalska, ME, Tajnik, M, Licastro, D, Bussani, E, Camparini, L, Mattioli, C et al. (2016). Therapeutic activity of modified U1 core spliceosomal particles. Nat Commun 7: 11168. 27041075 10.1038/ncomms11168 PMC4822034

[bib29] Tajnik, M, Rogalska, ME, Bussani, E, Barbon, E, Balestra, D, Pinotti, M et al. (2016). Molecular basis and therapeutic strategies to rescue factor IX variants that affect splicing and protein function. PLoS Genet 12: e1006082. 27227676 10.1371/journal.pgen.1006082 PMC4882169

[bib30] Bolton-Maggs, PH and Pasi, KJ (2003). Haemophilias A and B. Lancet 361: 1801–1809. 12781551 10.1016/S0140-6736(03)13405-8

[bib31] Giannelli, F, Green, PM, Sommer, SS, Poon, M, Ludwig, M, Schwaab, R et al. (1998). Haemophilia B: database of point mutations and short additions and deletions–eighth edition. Nucleic Acids Res 26: 265–268. 9399849 10.1093/nar/26.1.265 PMC147172

[bib32] Pollak, E and High, KA (2003) Genetic disorders of coagulation. In: Warrell, D, Cox, T, Firth, J, and Benz, E (eds) Oxford Textbook of Medicine, 4th ed., Vol. 3. Oxford University Press: Oxford, pp. 757–767.

[bib33] Hammond, SM and Wood, MJ (2011). Genetic therapies for RNA mis-splicing diseases. Trends Genet 27: 196–205. 21497936 10.1016/j.tig.2011.02.004

[bib34] Rogers, GL and Herzog, RW (2015). Gene therapy for hemophilia. Front Biosci (Landmark Ed) 20: 556–603. 25553466 10.2741/4324 PMC4476626

[bib35] Sabatino, DE, Nichols, TC, Merricks, E, Bellinger, DA, Herzog, RW and Monahan, PE (2012). Animal models of hemophilia. Prog Mol Biol Transl Sci 105: 151–209. 22137432 10.1016/B978-0-12-394596-9.00006-8 PMC3713797

[bib36] Dujardin, G, Lafaille, C, Petrillo, E, Buggiano, V, Gómez Acuña, LI, Fiszbein, A et al. (2013). Transcriptional elongation and alternative splicing. Biochim Biophys Acta 1829: 134–140. 22975042 10.1016/j.bbagrm.2012.08.005

[bib37] Rimessi, P, Fabris, M, Bovolenta, M, Bassi, E, Falzarano, S, Gualandi, F et al. (2010). Antisense modulation of both exonic and intronic splicing motifs induces skipping of a DMD pseudo-exon responsible for x-linked dilated cardiomyopathy. Hum Gene Ther 21: 1137–1146. 20486769 10.1089/hum.2010.010

[bib38] Pfaffl, MW (2001). A new mathematical model for relative quantification in real-time RT-PCR. Nucleic Acids Res 29: e45. 11328886 10.1093/nar/29.9.e45 PMC55695

