# Supplementary material for: An Exon-Specific U1snRNA Induces a Robust Factor IX Activity in Mice Expressing Multiple Human FIX Splicing Mutants
Source: Mol Ther Nucleic Acids. 2016 Oct 4;5(10):e370–. doi: 10.1038/mtna.2016.77 (PMC5095682; doi:10.1038/mtna.2016.77)
Supplement: Supplementary Figure S1 [file mtna201677x1.docx]

**A)**


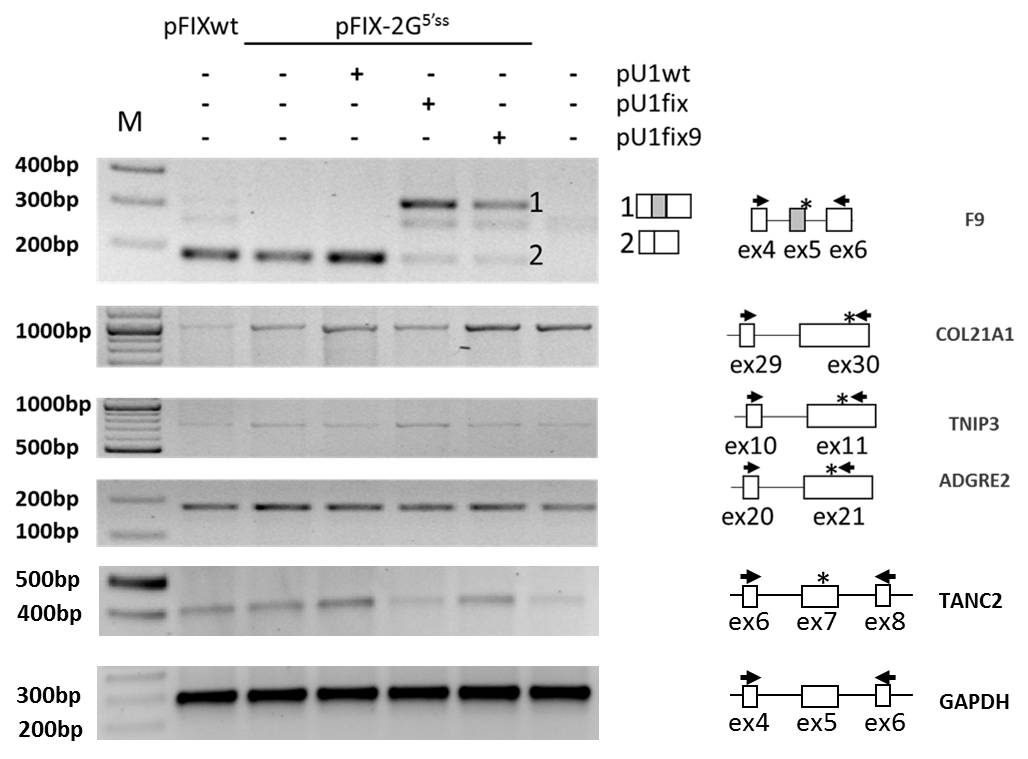


**B)**

| Gene name | Primer | Exon | Sequence 5’-3’ |
| --- | --- | --- | --- |
| ADGRE2 | For | 20 | ATCAGGAAATTGAAAACTGAGTCTG |
|  | Rev | 21 | AACAAAGTCTTTTCTTTCCCCTCTA |
| TNIP3 | For | 10 | CAGACTATCAGTGGTATGCTCTTGA |
|  | Rev | 11 | TGATCTGTCTAAGGAATAGCTTCCA |
| TANC2 | For | 6 | AGTAGATGAAAACATGACTGCTTCC |
|  | Rev | 7 | GTATGTCTCTGCTCATCCAGGTAAG |
| COL21A1 | For | 29 | GTTTGGGTATCCTGGAGAACAAG |
|  | Rev | 30 | AAAATGAAGGCATACAACAAACATT |
| GAPDH | For | 4 | TTTACATGTTCCAATATGATTCCAC |
|  | Rev | 6 | TTGTCATACTTCTCATGGTTCACAC |

***Supplementary Figure S1:***

A) Evaluation of alternative splicing patterns of putative off-target mRNAs in HEK293 cells transfected as indicated above. GAPDH exon 5 represents a constitutive exon and has been used as control. The schematic representation of the transcripts, of primers used for the RT-PCR (arrows), and of the position of the putative U1fix9 sequence targets by (asterisk) is reported in the right panel. Amplified products were separated on 2% agarose gel. M, 100 bp molecular weight marker.

B) Primers used to analyze U1fix9-potential off-targets and the GAPDH constitutive exon 5 as control.
